# Dissociating Task Selection and Response Selection in Dual-Task Contexts: Evidence from a Novel Trial-by-Trial Analysis of Temporal Overlap between Tasks

**DOI:** 10.5334/joc.485

**Published:** 2026-02-05

**Authors:** Patricia Hirsch, Iring Koch, Otmar Leo Bock

**Affiliations:** 1 Institute of Psychology, RWTH Aachen University, Aachen, Germany; 2 Institute of Exercise Training and Sport Informatics, Sport University, Cologne, Germany

**Keywords:** multitasking, dual task, task switching, task selection, response selection, cognitive control

## Abstract

This study examined the effect of temporal overlap in dual-task processing on task switch costs. Participants performed a psychological refractory period (PRP) experiment with a varying stimulus onset asynchrony (SOA), which is the time interval between the onsets of the stimuli for Task 1 (T1) and Task 2 (T2). Trials included task repetitions, where T2 was identical to T1, and task switches, where T2 differed from T1. T2 performance was worse with shorter SOAs than with longer SOAs and in switch trials than in repetition trials, indicating a PRP effect and switch costs. Notably, switch costs were not modulated by the SOA. However, SOA cannot precisely determine whether T1 and T2 are performed with or without temporal overlap in a given trial. To distinguish between these trials, we assessed the time interval between the T1 response and the onset of the T2 stimulus, known as the response-stimulus interval (RSI). The RSI acts as a proxy to temporally localize when T1 response selection is completed and the response-selection bottleneck is released, This novel trial-by-trial approach revealed that switch costs did not differ between trials with and without temporal overlap in task processing. Moreover, RSI was found to predicted T2 performance more accurately than SOA. Using RSI as a predictor of RT2 provides persuasive evidence that task selection and response selection rely on independent cognitive processes. Alternatively, both processes use shared central processing limitations, but temporal and/or strategic factors prevent these processes from overlapping in time and thereby interfering with each other.

Multitasking demands, such as switching between tasks or performing two tasks simultaneously, usually impair task performance (see, e.g., Koch et al., 2018, for a review). In task-switching contexts, performance costs are thought to occur at the level of task selection, whereas in dual-task settings, they are assumed to arise at the level of response selection. Although dual-task situations typically involve two different tasks and thus require task switching, little is known about the interaction between task-switching and dual-task manipulations. The present study aimed to bridge these two independent research lines by examining whether performance costs in task-switching situations vary as a function of the temporal overlap in task processing. From a theoretical perspective, this could help answer the question whether task selection and response selection rely on dependent cognitive processes.

## Multitasking: Empirical approaches and theoretical perspectives

During multitasking, several tasks are performed within a limited time period, causing the cognitive processes underlying the performance in these tasks to overlap in time (Koch et al., 2018). Such an overlap occurs, for instance, in task-switching and dual-task situations.

### Task-switching

Performance in situations with changing task demands is often examined using the task-switching paradigm (see, e.g., Kiesel et al., 2010; Vandierendonck et al., 2010, for reviews). In this paradigm, participants switch back and forth between tasks on a trial-by-trial basis. Performance is typically worse in switch trials than in repetition trials, resulting in *switch costs*. In switch trials, the task in a given trial differs from the task in the previous trial, whereas in repetition trials, the current task is identical to that in the previous trial.

*Task-set reconfiguration models* state that switch costs reflect cognitive control processes involved in task-set reconfiguration. It is theorized that executing a task presupposes the activation of its cognitive representation, termed task set, in working memory (e.g., Hirsch et al., 2016; Meiran, 2000; Rogers & Monsell, 1995; see Kiesel et al., 2010 for a review). In repetition trials, the previous task set can be reused, but in switch trials, the task set has to be reconfigured to match the new task demands, leading to switch costs.

In contrast, *task-set inertia accounts* propose that switch costs result from interference caused by the prior execution of other tasks (e.g., Allport et al., 1994; Allport & Wylie, 1999). That is, in switch trials, there is persisting activation of the unintended task set because it was needed in the previous trial. Additionally, there is persisting inhibition of the intended task set because it was irrelevant in the previous trial. This persisting activation and inhibition of task sets is detrimental to the performance in switch trials, resulting in switch costs.

Task-switching studies typically examine how task sets are maintained, updated, and switched, placing particular emphasis on task preparation and cognitive flexibility (see Koch et al., 2018, for a review). Thus, this research perspective primarily focuses on cognitive processes at the level of task selection.

### Dual tasking

Dual-task performance is often studied using the psychological refractory period (PRP) paradigm (see e.g., Hirsch & Koch, 2024; Pashler, 1994; Strobach, 2023, for reviews). In PRP trials, two stimuli are presented with a varying stimulus-onset asynchrony (SOA). Both stimuli are associated with a separate speeded categorization task, so that in each trial, participants perform two tasks—Task 1 (T1) and Task 2 (T2)—with a varying degree of temporal overlap. A typical finding is that reaction times in T2 (RT2) increase as the SOA decreases, indicating that temporal overlap in task processing impairs T2 performance (e.g., Bock et al., 2021; Jentzsch et al., 2007). This finding is referred to as the *PRP effect*.

To account for the PRP effect, researchers suggested that the processing of each task occurs in three serial processing stages: Stimulus perception, response selection, and response execution. The main idea is that the PRP effect arises because one of these stages cannot be devoted to two tasks simultaneously without a decline in performance. In several models, this processing stage refers to the central processes of decision and response selection (see, e.g., Fischer & Janczyk, 2022; Janczyk & Kunde, 2020, for reviews).

For instance, according to the *response-selection bottleneck model*, there is a structural capacity limitation at the response-selection stage, resulting in a processing bottleneck (e.g., Pashler, 1994). Due to this bottleneck, response selection for T2 can only begin after the response for T1 has been selected. RT2 increases with decreasing SOA because the waiting time for the bottleneck is longer with short SOAs than with long SOAs.

In contrast, *capacity sharing models* claim that response selection for T2 can be carried out in parallel with that for T1, but the limited central capacity at the response-selection stage has to be shared across the tasks (e.g., Tombu & Jolicoeur, 2003, see also Mittelstädt et al., 2022). Since short SOAs result in longer capacity-sharing periods than long SOAs, performance in T2 declines with decreasing SOA.

Finally, *strategic response-selection bottleneck models* state that the PRP effect is not attributable to structural processing limitations per se (e.g., Meyer & Kieras, 1997). Although response selection can proceed in parallel for two tasks, responses are selected serially to improve performance by preventing crosstalk between tasks (i.e., involuntary transmission of information across the processing streams of the tasks; e.g., Koch, 2009). For example, in the executive control of visual attention (ECTVA) model, serial response selection is ensured by activating the task set for T2 after response selection for T1 (Logan & Gordon, 2001).

So far, the majority of dual-task studies have aimed to locate the functional processing stage of dual-task bottlenecks (see Koch et al., 2018, for a review). Since several theoretical accounts propose the response-selection stage as the locus of the processing bottleneck, the primary focus of dual-task research has been on structural issues during response selection.

## Integrating task-switching and dual-task research

Task switching and dual tasks are among the most extensively studied forms of multitasking (Koch et al., 2018). However, the performance costs in these multitasking situations have typically been investigated within independent research lines and explained by paradigm-specific theoretical accounts (see ECTVA model by Logan & Gordon, 2001, for an exception). Methodological differences between the paradigms, along with the notion that switch costs and the PRP effect reflect different cognitive mechanisms (Pashler, 2000), might have contributed to the prevalent separation of these research lines.

### Differences and commonalities between task-switching and dual-task research

At the methodological level, the paradigms differ with regard to their key manipulations. In task-switching studies, temporal overlap in task processing is held constant because in each trial, only one stimulus is presented. Thus, participants always perform tasks sequentially. The key manipulation refers to the task sequence, and the performance is contrasted across task switches and task repetitions. This manipulation does not allow for measuring the effects of temporal overlap in task processing.

In contrast, in dual-task studies, two stimuli appear in each trial, and the key manipulation refers to the SOA which defines the degree of temporal overlap in the processing of two tasks. As participants typically perform different tasks as T1 and T2, the task sequence between T1 and T2 is held constant within a trial (e.g., Janczyk et al. 2018; Mattes et al., 2021). That is, there is always a task switch, and performance is contrasted across trials with strong temporal overlap (short SOA) and trials with less temporal overlap (long SOA). Hence, typical PRP studies do not allow for measuring switch costs.

In addition, there are paradigm-specific differences at the theoretical level (see Koch et al., 2018, for a review). Task-switching research has mainly focused on flexibility at the level of selecting task sets, whereas dual-task research has primarily examined structural issues related to processing bottlenecks during response selection (for exceptions: see e.g., Asuako et al. 2025; Hirsch et al., 2021; Kübler et al., 2022; Strobach et al., 2021). Thus, these research lines have addressed different processing levels.

However, in both research lines, there are two competing but not mutually exclusive ideas regarding the mechanisms of interference which pertain to bottom-up and top-down cognitive control (e.g., Worringer et al., 2019). Bottom-up processes are conceptualized as task-set inertia in task-switching models (e.g., Allport et al., 1994) and passive queuing of responses in dual-task models (e.g., Pashler, 1994). In contrast, top-down processes are conceptualized as task-set reconfiguration in task-switching research (e.g., Rogers & Monsell, 1995) and capacity sharing in dual-task research (e.g., Tombu & Jolicoeur, 2003). Thus, there are shared ideas about the sources of performance costs across the paradigms.

### Research on the interactions of task-switching and dual-task manipulations

Even though there is a task switch between T1 and T2 in typical PRP studies, the cognitive processes underlying this switch are rarely considered in models of dual-task interference. Hence it is not yet well understood how tasks are selected in dual-task contexts and whether there are dependencies between task selection and response selection.

To examine these questions, Hirsch, Nolden, Declerck et al. (2018) instructed participants to perform highly comparable dual-task and task-switching experiments. They found a correlation (*r* = .60) between the PRP effect in the dual-task experiment and switch costs in the task-switching experiment, indicating that the PRP effect increased as switch costs increased. The authors suggested that the correlation reflects shared underlying cognitive control processes.

However, the correlation might also reflect the role of unspecific cognitive processes, such as attention or short-term memory. To examine this alternative explanation, the dual-task experiment of Hirsch, Nolden, Declerck et al. (2018) also included a variation of the task sequence between T1 and T2. This manipulation resulted in switch trials, where T2 differed from T1, and repetition trials, where the same task was performed as T1 and T2. This task sequence manipulation was also used in studies by Band and van Nes (2006) and Lien et al. (2003). In all these studies, performance in T2 was worse in switch trials than in repetition trials, reflecting switch costs. Switch costs were not modulated by the SOA. Thus, the effect of the task sequence was independent of the effect of the SOA, supporting the aforementioned alternative view that the observed correlation between the PRP effect and switch costs might be related to shared unspecific cognitive processes rather than due to common underlying cognitive processes.

According to Band and van Nes (2006), additive effects of SOA and task sequence indicate that the task switch is deferred until the response selection for T1 is finished. This is because the waiting time for the availability of the response-selection bottleneck is not used to switch to T2, and therefore, the switch time fully contributes to RT2.

Beyond this additive pattern, there is also the theoretical possibility of an overadditive or underadditive interaction. In the case of an overadditive interaction, switch costs increase as SOA decreases. Such an effect could arise from lingering carry-over effects of task sets, as proposed in proactive interference models (e.g., Allport et al., 1994; see also Hirsch et al., 2015). Specifically, the activation of the T1 task set may decay over time, leading to reduced interference when switching to T2. At short SOAs, there is less time for this decay to occur, resulting in greater interference and, thus, larger switch costs compared to long SOAs.

In contrast, in the case of an underadditive interaction, switch costs are smaller with short than with long SOA. This pattern would suggest that the time during which T2 response selection waits for the availability for the response-selection bottleneck is used to initiate the switch to T2. Since the waiting time is longer with short SOAs than with long SOAs, switch costs get smaller with decreasing SOA. Thus, an underadditivity suggests that the T2 task set can already be activated before the response for T1 is selected.

Such an underadditive interaction was observed in a study by Hirsch et al. (2019), which used the same stimuli, tasks, responses, and SOAs as Hirsch, Nolden, Declerck et al. (2018), who did not find an interaction between SOA and task sequence. However, interpreting this underadditive interaction in Hirsch et al. (2019) is challenging, as it was observed only in the error rates for T2, which were lower with short SOAs than with long SOAs. This reversed PRP effects is highly unusual and rarely reported for T2 performance.

To sum up, most studies failed to find an interaction between SOA and task sequence (Band & van Nes, 2006; Hirsch, Nolden, Declerck et al., 2018; Lien et al., 2003), although there is evidence for a correlations between the PRP effect and switch costs (Hirsch, Nolden, Declerck et al., 2018). The notable exception is the interaction in the error rates for T2 observed by Hirsch et al. (2019) which is difficult to interpret due to the reversed PRP effect in the error pattern. This highlights the need for systematic replications, preferably with a wider range of SOAs, to clarify if and how response selection and task selection interact, and to gain a better understanding of the underlying cognitive processes.

## The present study

**Figure 1:**
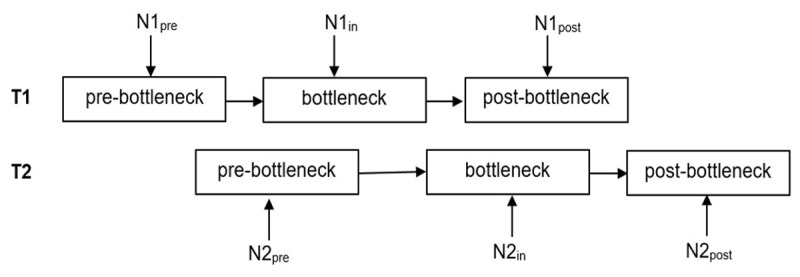
Pre-bottleneck, bottleneck, and post-bottleneck processing stages with noise (N) in Task 1 (T1) and Task 2 (T2).

To investigate whether switch costs are modulated by temporal overlap in task processing, prior studies have focused on the interaction of SOA and task sequence (e.g., Band & van Nes, 2006; Lien et al., 2003). However, the SOA-based approach assumes that the processing time for T1 and T2 is constant across trials. For example, if an SOA of 500 ms results in a temporal overlap of T1 and T2 processing in one trial, the SOA-based approach assumes that this particular SOA will result in a temporal overlap on all trials. Such a fixed-time view does not take the variability of central processing into account. That is, an SOA of 500 ms may result in T1–T2 temporal overlap on some trials but not on others, depending on stochastic variations in the speed of T1 and T2 processing. This is illustrated in Figure 1, where each processing stage is assumed to introduce noise.

**Figure 2:**
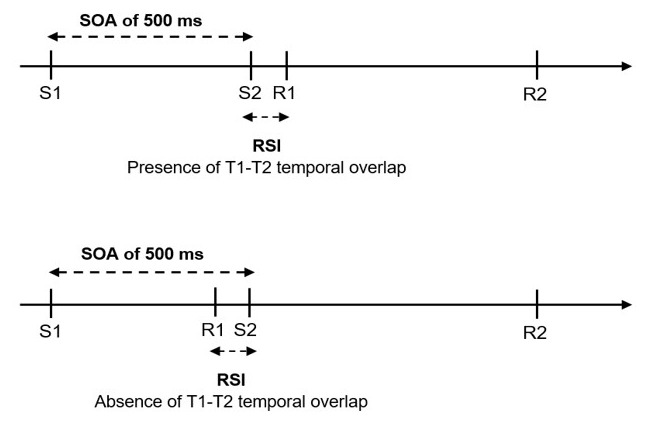
Two exemplary trials with SOA = 500 ms, where processing noise results in a temporal T1-T2 overlap on one trial (top part) but not on another trial (bottom part).

The aim of the present study was to examine the interplay between response selection and task selection using an approach that distinguishes between the presence or absence of T1 – T2 temporal overlap at the level of individual trials rather than at the SOA-level. To this end, we adopted an approach originally proposed by Vince and Welford (1967): Performance was analysed as a function of the time interval between the response to T1 and the onset of the T2 stimulus, referred to as the response-stimulus interval (i.e., RSI; see Figure 2). We reasoned that such an RSI-based analysis will allow us to distinguish more accurately between the presence of temporal overlap – characterized by a sharp increase of RT2 with decreasing RSI – and the absence of temporal overlap – characterized by a much smaller or even lacking dependence of RT2 on RSI.

As an additional difference to earlier studies, we employed a wider range of SOAs. Whereas prior studies used SOAs up to 900 ms, we employed SOAs up to 2,000 ms in order to yield a substantial number of trials without T1–T2 temporal overlap. Both methodological changes, RSI-based analysis and extended SOA range, allowed us not only to scrutinize the interplay between the PRP effect and switch costs on trials where both phenomena overlap, but also to determine if task switching is facilitated on trials without T1–T2 temporal overlap.

The first analysis followed standard procedures. That is, we analysed performance as a function of SOA and task sequence. We were mainly interested in the interaction of these variables.

The second analysis implemented the RSI-based approach. We classified trials with a strong RT2-RSI dependence as trials with temporal overlap in T1–T2 processing, and the remaining trials as those without such temporal overlap. We then used a linear mixed-effects model to determine the relationship between the dependent variable RT2 and the predictors RSI, sequence (repetition, switch) and trial class (overlap, non-overlap). We expected not only to confirm the outcome of the first analysis, but also to determine whether switch costs differ across trials with and without temporal overlap. If switch costs were higher with than without temporal overlap, this would suggest that the mere presence of T2 in a waiting queue increases the switch costs, irrespective of the waiting duration. If, however, switch costs were comparable with and without temporal overlap, this would suggest that the PRP effect and task switching are fully independent.

To evaluate the validity of the RSI-based approach, we replicated the second analysis using the same classification, but replacing the predictor RSI by SOA. This allowed us to compare the goodness of fit for the RSI-based and for the SOA-based approach.

### Method

#### Participants

Forty-four subjects (36 women; 24 right-handed; *M* = 22.32 years; *SD* = 2.59) with normal or corrected-to-normal vision and intact hearing participated in the experiment for partial course credit. All participants provided written informed consent before the experiment. The sample size was estimated for the effect of interest (i.e., interaction of SOA and task sequence) based on an a priori sample size calculation using G*Power (Faul et al., 2007). This calculation showed that 40 participants were needed to detect an effect of *f* = 0.23 with a power of .80. We used the effect size reported for the interaction effect in the study by Hirsch et al. (2019) and decided to test four additional participants to further increase power.

#### Stimuli, tasks, and responses

In addition to a tone of 600 Hz, the stimulus material consisted of a fixation cross (+), digits (i.e., 1 to 9, without 5), and letters (i.e., A, E, G, I, K, M, R, & U), all presented in a 40-point Arial font in white color on a black screen. Digits were linked to an odd vs. even categorization task (i.e., parity task) and letters to a consonant vs. vocal categorization task (i.e., letter task). Both categorization tasks could serve as T1 and T2. The stimuli for T1 always appeared to the left of the fixation cross, whereas the stimuli for T2 were presented to the right of the fixation cross. The fixation cross was presented in the center of the screen, and the distance between the stimuli and the fixation cross was 2 cm.

There were two horizontally arranged response key sets, each consisting of two keys. One key set was on the left side of the keyboard and included the y- and x-keys. The other key set was on the right side of the keyboard and comprised the n- and m-keys. Stimuli appearing to the left of the fixation cross required responses with the left key set and those appearing to the right of the fixation cross with the right key set. Both key sets could be used to complete the parity and letter tasks. The S-R mapping for a task was, however, constant across the key sets, meaning that the same spatial relations were used within the key sets (e.g., left key in both the left and right key set for ‘even’ and the right key in both key sets for ‘odd’). The S-R mapping for both tasks was counterbalanced across participants.

#### Procedure

Participants were tested individually in a single session that lasted approximately 45 minutes. At the beginning of the experiment, instructions were provided on the screen, emphasizing speed and accuracy for both tasks. First, participants, performed a practice block of 24 trials. After that, there were four experimental blocks with 65 trials each.

Each trial started with a simultaneous presentation of the tone and the fixation cross. After 100 ms, the stimulus for T1 appeared to the left of the fixation cross. The stimulus for T2 was presented after a random SOA of 50 ms, 450 ms, 1,000 ms, or 2,000 ms. Immediately after the response to T2, the stimuli for both tasks disappeared. The inter-trial interval was 2,900 ms and resulted in a pairwise organization of T1 and T2.

There were repetition trials, where the same task was performed as T1 and T2, and switch trials, where T2 differed from T1. These trial types were presented randomly, with the stipulation that an equal number of repetitions and switches was included within a block.

#### Statistical analysis

As a first step, we employed a classical approach to examine the effect of SOA on switch costs. T1 and T2 performance was analysed using a 4 × 2 repeated-measures design with the independent within-subject variables *SOA* (50 ms, 450 ms, 1,000 ms, vs. 2,000 ms) and *task sequence* (switch vs. repetition). The dependent variables were RTs and error rates, which were analyzed with separate analyses of variance (ANOVAs). When the assumption of sphericity was violated, we applied a Greenhouse-Geisser correction to adjust the *F*-values.

The second step of analysis implemented the RSI-based approach in the R software environment. Since the relationship between RT2 and RSI exhibited a distinct breakpoint (see Figure 5), we identified the RSI-value of this breakpoint using segmented linear regression (functions *lm* and *segmented*). This was done separately for trials with a repetition sequence and those with a switch sequence, considering that breakpoints might differ between sequences. Data were then classified as having temporal overlap in T1–T2 processing if their RSI was smaller than the breakpoint, and were classified as having no such overlap otherwise.

We subsequently analyzed RT2 with a linear mixed-effects model (LMM) using the R function *lmer*. The model structure was


1 \[
\mathrm{RT2\sim \left(sequence+RSI\right)*class+\left(1|participant\right)}
\]

In this model, RSI is a continuous fixed effect, sequence (repetition, switch) and class (overlap, non-overlap) are categorical fixed effects, and participant is included as a random intercept. This model let us to determine whether the RT2 – RSI slope differed between trials with and without overlap (RSI x class interaction), whether switch costs were present (main effect of sequence), and whether switch costs differed between trials with and without overlap (sequence x class integration).

The overall contribution of each effect was tested using Type II Wald chi-square tests (function *Anova*). Following established procedures (Field et al., 2012), effect sizes were expressed as *d* = (estimated effect/residual standard deviation), with d = 0.2/0.5/0.8 indicating a small/medium/large effect.

Since LMMs are based on individual observations rather than aggregate means, they process a large number of data (9614 in our study). Statistical significance can therefore be yielded even if effect sizes are negligible. We therefore adopted recommendations in literature (e.g., Lantz, 2013) to focus on the combination of statistical significance *and* non-negligible effect size, often referred to as ‘practical’ or ‘substantive’ significance. Thus, we defined substantive significance as *p* < .05 combined with *d* > 0.2.

To examine whether RSI is indeed a justifiable predictor of T2 performance, we re-calculated (1) using trial-level SOA rather than RSI as a predictor, and compared the fits of both models using three established model selection criteria (e.g., Nakagawa & Schielzeth, 2013): AIC (Akaike information criterion, function *AIC*), BIC (Bayesian information criterion, function *BIC*), and marginal R^2^ (fixed-effect variance, function *r2*). The alternative approach, to calculate a model with both RSI and SOA as predictors, was precluded by multicollinearity: Generalized variance inflation factors (function *vif*) exceeded the conventional threshold of 10, reaching values up to 71.8.

Finally, we analyzed response accuracy with model structure (1). However, since trial-level accuracy is a binary variable, with each response being either correct or incorrect, this analysis was implemented as a generalized linear mixed-effects model (R function *glmer* with a binomial distribution and logit link). The overall contribution of each effect was assessed by Type II Wald chi-square tests. Effect sizes were calculated following Chinn (2000) as *d* = (estimated log-odds /1.81).

All analyses excluded all practice trials and the first trial in each block. For the RT analyses, we additionally discarded trials with RTs deviating more than –/+ 3 *SD* from each individual’s mean per condition and trials with an error in T1 and/or T2.

## Results

### ANOVA: Performance as a function of SOA and task sequence

*T1*

**Figure 3:**
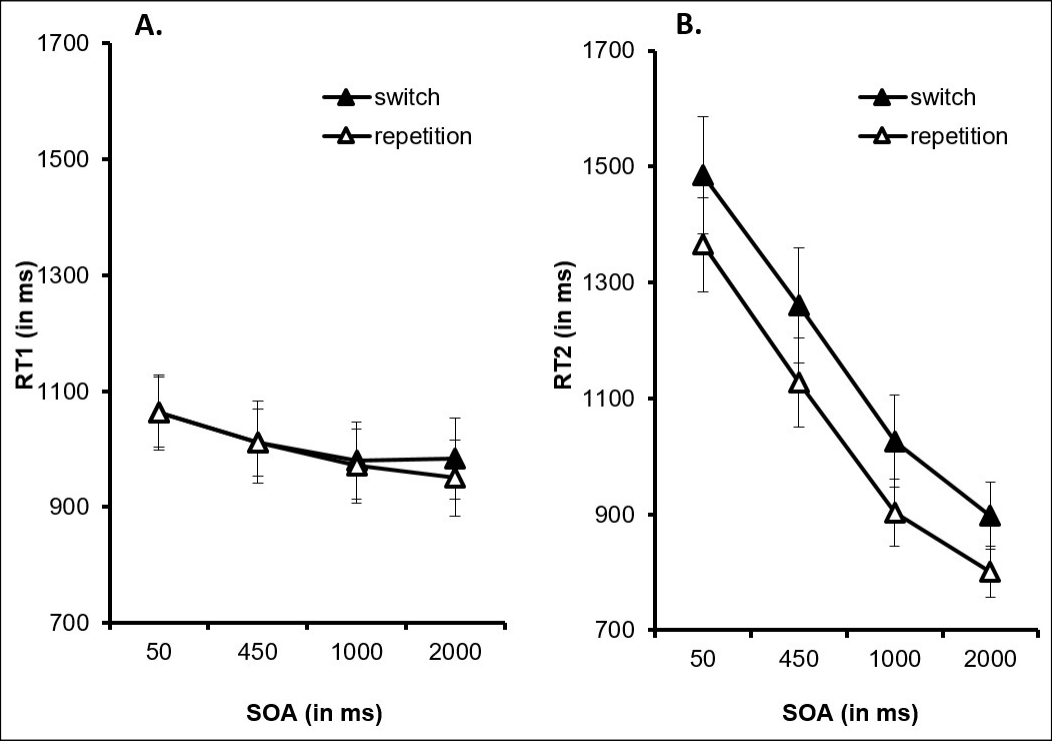
**A.** Reaction times for Task 1 (T1, RT1) and **B.** reaction times for Task 2 (T2, RT2) as a function of the stimulus onset asynchrony (SOA) and of the task sequence. Error bars represent the standard error of the mean.

The ANOVA on RT1 showed a main effect of *SOA, F*(1.64, 70.44) = 9.318, *p* < .001, η_p_^2^ = .178, reflecting decreasing RT1 with increasing SOA (1063 ms, 1011 ms, 975 ms, vs. 967 ms; see Figure 3A). A post-hoc analysis revealed significantly longer RT1 with an SOA of 50 ms than with an SOA of 450 ms (mean difference: 52 ms; *p* = .019, 95%-CI [5.99–97.89]), an SOA of 1,000 ms (mean difference: 88 ms; *p* = .001, 95%-CI [27.87–148.09]), and an SOA of 2,000 ms (mean difference: 97 ms; *p* = .014, 95%-CI [14.06–178.99]). In addition, RT1 was significantly longer with an SOA of 450 ms than with an SOA of 1000 ms (mean difference: 36 ms; *p* = .04, 95%-CI [1.12–70.96]). The main effect of *task sequence* and the interaction of *SOA* and *task sequence* were non-significant, both *F*s < 1 and *p*s > .391.

**Figure 4:**
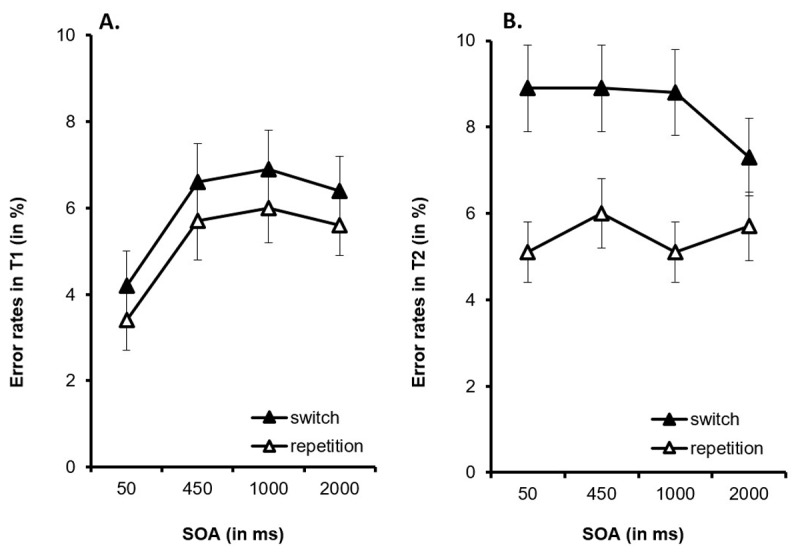
**A.** Error rates in Task 1 (T1) and **B.** in Task 2 (T2) as a function of the stimulus onset asynchrony (SOA) and of the task sequence. Error bars represent the standard error of the mean.

For the accuracy data, the main effect of *SOA* was significant, *F*(3, 129) = 6.25, *p* < .001, η_p_^2^ = .127 (3.8%, 6.1%, 6.5%, vs. 6.0%, see Figure 4A). A post-hoc analysis demonstrated significantly lower error rates with an SOA of 50 ms than with an SOA of 450 ms (mean difference: 2.3%; *p* = .021, 95%-CI [0.2–4.4]), an SOA of 1,000 ms (mean difference: 2.7%, *p* < .001, 95%-CI [1.1–4.2]), and an SOA of 2,000 ms (mean difference: 2.2%; *p* = .006, 95%-CI [0.5–3.9]). The main effect of *task sequence, F*(1, 43) = 3.058, *p* = .087, η_p_^2^ = .066, and the interaction of *SOA* and *task sequence* were not significant, *F* < 1.

*T2*

For RT2, there was a significant main effect of *SOA, F*(1.37, 58.92) = 121.74, *p* < .001, η_p_^2^ = .739. RT2 decreased as SOA increased, reflecting a PRP effect (1425 ms, 1194 ms, 964 ms, vs. 849 ms; see Figure 3B). A post-hoc analysis revealed that RT2 was longer with an SOA of 50 ms than with an SOA of 450 ms (mean difference: 231 ms; *p* < .001, 95%-CI [179–283]), an SOA of 1,000 ms (mean difference: 461 ms; *p* < .001, 95%-CI [375–547]), and an SOA of 2,000 ms (mean difference: 576 ms; *p* < .001, 95%-CI [443–708]). In addition, RT2 was longer with an SOA of 450 ms than with an SOA of 1,000 ms (mean difference: 230 ms; *p* < .001, 95%-CI [163–297]) and an SOA of 2,000 ms (mean difference: 345 ms; *p* < .001, 95%-CI [231–458]). Finally, RT2 was longer with an SOA of 1,000 ms than with an SOA of 2,000 ms (mean difference: 115 ms; *p* < .001, 95%-CI [50–179]).

Moreover, the main effect of *task sequence* was significant, *F*(1, 43) = 20.006, *p* < .001, η_p_^2^ = .318. Participants responded more slowly in switch trials than in repetition trials (1167 ms vs. 1049 ms), resulting in switch costs of 118 ms. Most importantly, the interaction of *SOA* and *task sequence* was not significant, *F* < 1.

For the accuracy data, the ANOVA also yielded a main effect of *task sequence, F*(1, 43) = 15.864, *p* < .001, η_p_^2^ = .269, with more errors in switch trials than in repetition trials (8.5% vs. 5.5%; see Figure 4B), reflecting switch costs of 3.0%. All other effects were not significant, both *F*s < 1.266 and *p*s > .289.

*Summary*

For T1, RTs decreased with increasing SOA, while error rates increased with increasing SOA, at least between SOAs of 50 ms to 450 ms.^1^ The pattern in T1 suggests that participants may have adopted a more cautious processing strategy for T1 when anticipating temporal overlap with T2. For T2, RTs decreased with increasing SOA, reflecting the expected PRP effect. Moreover, RTs were longer and error rates were higher for T2 in switch trials than in repetition trials, resulting in switch costs. Most importantly, switch costs were not affected by the SOA.

### Linear mixed-effect models: RT2 as a function of RSI

A disadvantage of the ANOVA approach is the need to average across trials which makes it impossible to distinguish between trials with temporal overlap in T1 and T2 processing and those without temporal overlap. Thus, the data in Figure 3B are compatible with the view that the PRP effect lasted *less than 2000 ms*, such that RT2 scores at SOA = 2000 ms are trials without T1–T2 temporal overlap. However, the data are also compatible with the view that that the PRP effect lasted *more than 2000 ms* such that the RT2 scores at SOA = 2000 ms are from trials with T1–T2 temporal overlap. Finally, the data are also compatible with the view that the PRP effect lasted *close to 2000 ms* such that the RT2 scores at SOA = 2000 ms form a mix of these two trial categories.

**Figure 5:**
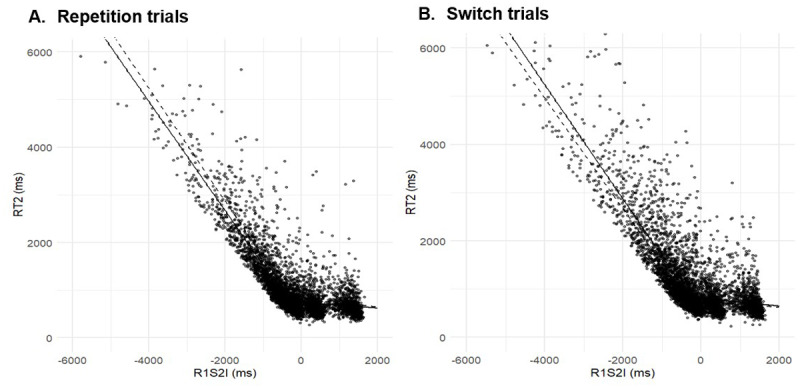
Reaction times for Task 2 (RT2) as a function of the response-stimulus interval (RSI). Data are plotted separately for repetition trials (left) and switch trials (right). Solid lines represent segmented linear regression fits to the data points in the respective graph. For comparison, dashed lines show the corresponding fits from the other graph.

To overcome this problem, our second analysis was performed at trial-level rather than at SOA-level. Figure 5 illustrates that the relationship between RT2 and RSI exhibited a distinct breakpoint, both for repetition trials and for switch trials. The location of this breakpoint was determined by segmented linear regression, yielding RSI = –339.82 ms (SE = 13.71) for repetition trials and RSI = 280.98 ms (SE = 17.86) for switch trials. The difference between these estimates was 59.29 ms, (95% CI [–26.65, 119.23], d = 2.61). Since the confidence interval included zero, we found no evidence for breakpoints to differ between the two sequences, and we therefore adopted the mean of both breakpoints, RSI = 310.4 ms, to classify trials a being with or without overlap.

The LMM outcome for the dependent variable RT2 is summarized in Table 1. Substantive significance was found for RSI (larger RT2 at smaller RSI), class (larger RT2 on switch than on repetition trials), sequence (larger RT2 left of the breakpoint), and RSI:class (steeper slope left of the breakpoint), but not for sequence:class (similar sequence effect to the left and to the right of the breakpoint).

**Table 1:** Results of the linear mixed-effects model for the dependent variable RT2. Substantive significance (*p* < 0.05 combined with *d* > 0.2) is indicated in bold. *d* > 0.2, *d* > 0.5 and *d* > 0.8 is regarded a small, medium and large effect size, respectively.


	χ^2^	df	*p*	*d*

RSI	7723.7	1	**<0.001**	**1.620**

Class	21.3	1	**<0.001**	**0.554**

sequence	232.7	1	**<0.001**	**0.332**

RSI:class	7929.5	1	**<0.001**	**2.940**

sequence:class	47.5	1	<0.001	0.031


*Comparing linear mixed effects models with RSI versus SOA as a predictor*

To determine whether using RSI instead of SOA as a predictor is justifiable, goodness-of-fit metrics were calculated for the above LMM and for a control LMMs in which RSI was replaced by SOA. Since SOA has been the traditional predictor throughout the history of PRP research, it may have a closer causal relationship to RT2 than RSI. We thus yielded AIC = 139305, BIC = 139362 and marginal R^2^ = 0.706 for the RSI-based model, compared to AIC = 150047, BIC = 150104 and marginal R^2^ = 0.083 for the SOA-based model. Thus, all three metrics indicate a better fit of the RSI-based model compared to the SOA-based model. Furthermore, the confidence interval for the difference between both marginal R^2^ (95%CI [0.64, 0.60]) did not include zero, thus supporting the robustness of the RSI advantage.

To examine the generalizability of the advantage of using RSI as a RT predictor, we replicated the model comparison for a different set of RT2 data available in our laboratory (Hirsch, Nolden, Philipp et al., 2018). It included 11184 observations from 48 participants, and did not involve sequence as a predictor. Using the same processing pipeline described above, this data set yielded a breakpoint at RSI = –387; goodness of fit for the RSI-based model was AIC = 160455, BIC = 160499 and marginal R^2^ = 0.754; goodness of fit for the SOA-based model was AIC = 170026, BIC = 170070 and marginal R^2^ = 0.214 for the SOA-based model. Thus again, the RSI-based model fitted the data better than the SOA-based model. The confidence interval for the difference between the two marginal R^2^ (95%CI [0.64, 0.60]) did not include zero, again supporting the robustness of the RSI advantage.

*Linear mixed-effect models: Accuracy as a function of RSI*

The generalized LMM outcome for accuracy as the dependent variable is shown in Table 2. Substantive significance was found only for sequence (mean error rate 5.5% on repetition trials, (8.5% on switch trials), thus replicating the outcome of classical ANOVA described above.

**Table 2:** Results of the linear mixed-effects model for accuracy as the dependent variable. Substantive significance (*p* < 0.05 combined with *d* > 0.2) is indicated in bold.


	χ^2^	df	*p*	*d*

RSI	0.17	1	0.681	0.014

Class	0.21	1	0.645	0.016

sequence	35.61	1	**<0.001**	**0.270**

RSI:class	3.32	1	0.068	0.124

sequence:class	2.85	1	0.092	0.194


*Summary*

The breakpoint between trials with and without T1–T2 temporal overlap was comparable for repetition and switch trials. Switch costs were present (i.e., effect of sequence), and did not differ between trials with and without overlap. RSI was a better predictor of RT2 compared to the traditionally used predictor SOA in the present data, and also in another available data set.

## Discussion

The aim of the present study was to examine whether switch costs in dual-task contexts are affected by temporal overlap in task processing. To this end, participants performed a PRP experiment in which T2 required a task switch on some trials, and a task repetition on other trials. In agreement with previous findings, we observed a PRP effect and switch costs in T2. Moreover, we confirmed that switch costs were not affected by SOA. Since an analysis with SOA as the dependent variable does not separate well between trials with and without temporal overlap in task processing (particularly for medium SOAs, where some trials might involve temporal overlap, while other do not), we additionally performed a trial-level analysis where the dependent variable was RSI (i.e., the time interval between T1 response and T2 onset). This allowed us to distinguish between trials with and without temporal overlap in T1–T2 processing, to document for the first time that switch costs did not differ between trials with and without overlap, and to establish RSI as a novel and more robust predictor of RT2 compared to SOA. Furthermore, we provided additional support for the latter conclusion by analysing another set of data about the PRP effect.

### Effects of the T1–T2 sequence

As expected from the literature, performance in T2 was worse in switch trials than in repetition trials. Thus, we replicated switch costs with SOAs in a wider range than those used in previous studies. The presence of switch costs suggests that in dual-task contexts in which T2 differs from T1, information required for the processing of T1 is represented separately from information needed to perform T2 (see Hirsch & Koch, 2024, for a discussion on dual-task representations). As a consequence, time-consuming and error prone reconfiguration processes are needed in switch trials but not in repetition trials where the task set relevant for T1 can be reused for T2 (Meiran, 1996). Additionally, proactive interference between task sets might contribute to switch costs in T2 (e.g., Allport et al., 1994; Allport & Wylie, 1999).

### Effects of temporal overlap in task processing

Besides switch costs, we observed a PRP effect (e.g., Pashler, 1994), indicating that T2 performance is worse with short SOAs than with long SOAs. Note, however, that there was also a SOA effect in T1. Whereas RT1 was longer with short compared to long SOAs, error rates in T1 were lower with short SOAs than with long SOAs. This data pattern suggests that participants may have adopted a more cautious processing strategy for T1 when anticipating temporal overlap with T2.

Crucially, despite the effect of SOA on RT1, we observed a robust PRP effect across all SOAs, and the PRP effect in T2 was substantially larger than that in T1. It is therefore possible that a small portion of the PRP effect in T2 reflects a propagation of the SOA effect in T1. This notion is also supported by findings from a study by Pashler and O’Brian (1993). They examined the relationship between the speed of the T2 response and that of the corresponding T1 response by subdividing RT1 into quintiles and investigating whether RT2 differed as a function of the RT1 quintile. Interestingly, Pashler and O’Brian (1993) observed that RT2 increased with increasing RT1, but this effect occurred primarily for short SOAs where both tasks overlapped in time, indicating that SOA effects in T1 can propagate to T2. However, since in the present study, the PRP effect in T2 was much larger than the SOA effect in T1, the majority of the PRP effect in T2 cannot be accounted for by such a propagation mechanism, suggesting that the SOA affected T2 processing itself. Taken together, it can thus be concluded that the central bottleneck characteristic of dual-task performance was preserved and that our key findings remain valid and interpretable.

The SOA is typically manipulated to induce more or less temporal overlap in task processing. However, SOA does not separate between trials with and without temporal overlap in task processing. To get more systematic insights into the effect of temporal overlap in task processing on switch costs, we also analysed trial-level performance as a function of RSI.

This analysis revealed that the relationship between RSI and RT2 can be approximated by a piecewise linear function, with a breakpoint at an RSI of about –300 ms. Hence, on trials with RSI < breakpoint, the stimulus for T2 appeared before the response in T1 was executed. On trials with RSI > breakpoint, however, the T2 stimulus appeared after the T1 response. We observed a strong inverse relationship between RT2 and RSI below the breakpoint, which tapered off above the breakpoint; this documents the existence of a PRP effect even when RSI rather than SOA is adopted as the dependent variable. In the language of the central bottleneck theory, our findings indicate that T1 leaves the response-selection bottleneck about 300 ms before the T1 response is executed. The interval between leaving the bottleneck and generating an overt response is probably needed to assign the response decision to a specific finger, to generate the corresponding motor program, and to overcome biomechanical delays, in accordance with the motor execution phase of typical PRP models (e.g., Pashler, 1994).

Another important finding is that RSI is a better predictor of RT2 compared to SOA, indicating for the first time that the waiting time for T2 to enter the response-selection bottleneck depends more strongly on when T1 response selection is finished, rather than when the T1 stimulus is presented. One possible explanation for this finding is that in contrast to the SOA analysis, the RSI analysis takes into account the noise in T1 processing. When subdividing this noise into three components—pre-bottleneck noise, bottleneck noise, and post-bottleneck noise—the RSI-based analysis incorporates all of them (see Figure 1), while the SOA-based analysis incorporates none. Since the waiting time for T2 before the bottleneck is only influenced by the first two components, it appears that the sum of pre-bottleneck noise and bottleneck noise is greater than post-bottleneck noise, so that the RSI-based analysis is superior to the SOA-based analysis.

Note, however, that although RSI provides a useful trial-by-trial measure of temporal overlap, it can be influenced by response-grouping strategies (see Ulrich & Miller, 2008, for response-grouping accounts). These strategies can artificially inflate RT1 and may lead to an overestimation of temporal overlap in T1 and T2 processing. In trials with response grouping, participants select the response for T1 but delay its execution in order to produce it in close succession with the response for T2. As a result, RT1 may increase with decreasing SOA, leading to negative RSI values, even if actual temporal overlap in T1 and T2 processing is absent. However, in our study, participants were explicitly instructed not to delay their T1 response until the onset of the T2 stimulus or the response for T2 and trials involving response grouping, as indicated by inter-response intervals (i.e., IRT < 100 ms), were rare.^2^ Nonetheless, this potential limitation should be considered when interpreting RSI effects.

### Effects of temporal overlap in task processing on switch costs

The SOA analysis revealed that switch costs were comparable in size across a wide range of SOAs, possibly including SOAs with and without temporal overlap in task processing. This finding is consistent with the studies by Band and van Nes (2006), Hirsch, Nolden, Declerck et al. (2018), and Lien et al. (2003), but not in line with the study by Hirsch et al. (2019) who observed smaller switch costs with a short SOA than with a long SOA.

However, this interaction was observed in the error rates, which were smaller for short SOAs than for long SOAs. Due to this reversed PRP effect, the interaction is difficult to interpret. This unusual error pattern may reflect a different processing strategy adopted by participants in that particular study (see e.g., Miller et al., 2009, for processing strategies). Notably, studies that did not observe this atypical SOA effect in the error rates also did not report an interaction. This suggests that the interaction found in Hirsch et al. (2019) may be specific to the particular strategic context in that study rather than reflecting a generalizable cognitive phenomenon.

Importantly, it is problematic to distinguish between trials with and without temporal overlap based on SOA, and more clarity is provided when trials are categorized based on RSI (see Introduction): piecewise linear regression of RT2 on RSI yielded a breakpoint at about RSI = –310 ms, allowing us to categorise trials with a lower RSI as trials with temporal overlap in T1 and T2 processing, and trials with a higher RSI > as those without temporal overlap in task processing.

The present study showed for the first time that switch costs did not differ between trials with and without temporal overlap in task processing. This finding does not align with the view that response selection in the bottleneck requires a constant quantity of processing resources which, therefore, are not available for task switching. If this were the case, switch costs should be higher on trials with temporal overlap in T1 and T2 processing compared to trials without temporal overlap.

Summing up, our findings from both the RSI-analysis and the SOA-analysis indicate that switch costs and the performance decline due to temporal overlap in task processing rely on distinct cognitive control processes that do not draw on the same central processing capacity. Second, it is also possible that both effects stem from shared central processing limitations, but that temporal and/or strategic factors prevent these processes from overlapping and thereby interfering with each other. For example, there may be a structural limitation that allows central capacity to be allocated to only one process at a time, either to response selection in T1 or to task-set reconfiguration in T2. Alternatively, participants may strategically postpone task-set reconfiguration until response selection in T1 is completed or reached a stage that can occur in parallel with task-set reconfiguration for T2, in order to reduce interference between processes. In the framework of the response-selection bottleneck, this can be explained by assuming that task-set reconfiguration is located after the response-selection bottleneck (see Lien et al., 2003, for a similar idea). This is also consistent with the ECTVA model (Logan & Gordon, 2001), which posits that task-set reconfiguration takes place following the selection of the T1 response.

Note, however, that Band and van Nes (2006) proposed that there are different sources of switch costs, including the activation of task sets (e.g., Rogers & Monsell, 1995), retrieving task sets from long-term memory (e.g., Mayr & Kliegl, 2000), and inhibiting old task sets (e.g., Mayr & Keele, 2000). According to Band and van Nes (2006), temporal overlap might have opposite effects on these processes. Therefore, more research is needed to decompose these processes.

Finally, it has to be noted that PRP studies typically employ highly different tasks for T1 and T2 in order to minimize crosstalk and to maximize the possibility to perform tasks in parallel. However, in studies investigating the interaction between task sequence and SOA, repetition trials involve the same task in both T1 and T2. This opens the possibility that unlike in switch trials, the perceptual processing of the stimulus for T2 may be postponed until the response selection for T1 is completed, potentially as a strategy to avoid crosstalk. This raises a general concern in PRP studies that examine the interplay of SOA and task-sequence effects because it questions whether parallel processing occurs in repetition trials with short SOA.

In the present study, however, it was ensured that in repetition trials with short SOA, at least some degree of parallel processing occurred.^3^ This is essential for interpreting the results, as it ensures that any differences between switch and repetition trials could be meaningfully attributed to cognitive control mechanisms.

### Summary and conclusions

In the present study, we used RSI as a proxy to temporally localize when response selection in T1 is completed and, thus, when the response selection bottleneck is released. This information cannot be obtained when using SOA as a predictor of RT2. By employing RSI, trials can be clearly categorized as involving or not involving temporal overlap in task processing. The present study showed that the presence of a temporal overlap in T1 and T2 processing does not affect switch costs. This finding indicates that task selection and response selection in dual-task settings rely on independent cognitive processes, or alternatively both effects stem from shared central processing limitations, but that temporal and/or strategic factors prevent these processes from overlapping and thereby interfering with each other. Based on the findings of the present study, RSI appears to be a more appropriate predictor of T2 than SOA in dual-task research, given its stronger relationship with RT2. Nonetheless, future studies are required to determine whether the RSI is indeed a better predictor of RT than SOA.

## Data Accessibility Statement

Raw data is publicly available on PsychArchives. https://doi.org/10.23668/psycharchives.21609.

## Footnotes

To analyse potential speed-accuracy trade-offs, we computed balanced integration scores (BIS) as integrated measures of speed and accuracy, following the approach proposed by Liesefeld and Janczyk (2023), and repeated the ANOVA for T1. Consistent with the ANOVAs on RTs and error rates, the main effect of *task sequence, F*(1, 43) = 3.764, *p* = .059, η_p_^2^ = .08, and the interaction of *SOA* and *task sequence* were not significant, *F* < 1. In contrast to the ANOVAs on RT and error rates, the main effect of *SOA* was no longer significant, *F*(1, 43) = 1.645, *p* = .206, η_p_^2^ = .037. The disappearance of the aforementioned SOA effects in T1 when using BIS might indicate that those T1 effects may have reflected a shift in the speed-accuracy tradeoff from slower and more accurate responses at low SOAs to faster and less accurate responses at high SOAs. Note, however, that the focus of the present study is on T2.

To assess whether response grouping may have affected our results, we calculated inter-response times (IRTs) for all trials, following the recommendations outlined by Ulrich and Miller (2008). Trials with IRTs below 100 ms were considered indicative of response grouping. There were only 0.741% trials with this IRT. We excluded these trials and re-ran all relevant ANOVAs. The results remained unchanged.

To examine if participants only initiated perceptual processing in T2 after completing response selection in T1, we investigated whether characteristics of T2 influenced performance in T1. To this end, we categorized trials as compatible or incompatible. A trial was defined as compatible when the responses for T1 and T2 overlapped at a spatial level. That is, when both required a left-finger response (*y* in T1 and *n* in T2) or both required a right-finger response (*x* in T1 and *m* in T2). In contrast, a trial was categorized as incompatible when T2 required a spatially different response than T1 (*y* in T1 and *m* in T2, or *x* in T1 and *n* in T2). One-tailed *t*-tests on T1 performance in repetition trials with a SOA of 50 ms showed that RT did not significantly differ as a function of compatibility (t < 1), but error rates were significantly higher in incompatible than in compatible trials (*t*(43) = 2.24, *p* = .015, *d* = 0.34). This backward-compatibility effect indicates that participants had already begun processing T2 while still selecting the response for T1 (Hommel, 1998), suggesting that at least some degree of parallel processing occurred, even in repetition trials with strong crosstalk and short SOAs. This finding supports the view that T2 was not entirely postponed until T1 was completed, and thereby strengthens the interpretability of the results.
